# Clinical Characteristics, Infection Profiles, and Hospital Outcomes of Intensive Care Unit Patients Undergoing Antimicrobial Therapy with Ceftolozane/Tazobactam: A Multicentric Retrospective Analysis

**DOI:** 10.3390/antibiotics15040382

**Published:** 2026-04-09

**Authors:** Pedro Kurtz, Pedro F. Del Peloso, Arthur M. Albuquerque, Bianca B. P. Antunes, Grazielle V. Ramos, Fernando A. Bozza

**Affiliations:** 1 Dor Institute of Research and Education, Rio de Janeiro 22281-100, Brazil; kurtzpedro@mac.com (P.K.); grazielle.viana@idor.org (G.V.R.); 2 Intensive Care Department, Instituto Estadual do Cérebro (IECPN), Rio de Janeiro 20231-092, Brazil; 3 Richet Laboratory, Rio de Janeiro 20231-092, Brazil; pedro.peloso@richet.com.br; 4 School of Medicine, Universidade Federal do Rio de Janeiro, Rio de Janeiro 21941-971, Brazil; arthur_albuquerque1@yahoo.com; 5 Department of Industrial Engineering (DEI), Pontifical Catholic University of Rio de Janeiro (PUC-Rio), Rio de Janeiro 22451-900, Brazil; biancabrandao@tecgraf.puc-rio.br; 6 Instituto Nacional de Infectologia Evandro Chagas (INI), FIOCRUZ, Rio de Janeiro 21040-360, Brazil

**Keywords:** multidrug-resistant, ceftolozane/tazobactam, Gram-negative, antimicrobial therapy

## Abstract

**Introduction**: Ceftolozane/tazobactam is a β-lactam/β-lactamase inhibitor with potent activity against multidrug-resistant Gram-negative pathogens, notably *Pseudomonas aeruginosa*. Its use is vital in the management of severe infections in ICU patients, especially where resistance limits first-line antibiotic options. This study aimed to describe the clinical characteristics, infection profiles, antimicrobial therapies, and hospital outcomes of ICU patients in Brazil who were treated with ceftolozane/tazobactam. **Methods**: A multicenter retrospective analysis was conducted on ICU patients treated with ceftolozane/tazobactam across twelve private hospitals in Brazil admitted between July 2018 and February 2023. Data were extracted from electronic medical records, including demographics, comorbidities, infection characteristics, antimicrobial usage, and hospital outcomes. Additionally, microbiological data and treatment details were evaluated. Descriptive statistics were used for analysis. **Results**: The study included 104 patients, with a median age of 78 years (IQR 61, 87) and 43% of whom were female. Pneumonia (57%) and urinary tract infections (19%) were the primary indications for treatment. Mechanical ventilation was required in 62 patients (60%). Pathogens were isolated in 44 cases, with ESBL-producing Gram-negative bacteria (34%) and carbapenem-resistant strains (18%) being the most common. Empirical therapy was initiated in 57% of cases, with a median treatment duration of 10 days. Concomitant antibiotics were used in 64% of patients. Mechanically ventilated patients exhibited higher mortality (66% vs. 29%) and prolonged ICU stays (57 vs. 20 days) as compared to non-ventilated patients. **Conclusions**: Ceftolozane/tazobactam demonstrates efficacy and safety in managing multidrug-resistant Gram-negative infections in ICU settings. However, its empirical use, driven by high resistance rates, underscores the importance of microbiology-guided therapy. The observed high mortality and prolonged ICU stay highlight the critical need for optimized infection management strategies and continued antimicrobial stewardship in critically ill populations.

## 1. Introduction

Ceftolozane/tazobactam is a β-lactam/β-lactamase inhibitor combination with potent activity against Gram-negative pathogens, including multidrug-resistant (MDR) strains. It is primarily utilized for the treatment of complicated urinary tract infections (cUTIs), including pyelonephritis; complicated intra-abdominal infections (cIAIs), in combination with metronidazole; and hospital-acquired or ventilator-associated bacterial pneumonia (HABP/VABP) [1,2,3,4]. The spectrum of ceftolozane/tazobactam makes it an important agent for treating infections caused by pathogens such as extended-spectrum β-lactamase (ESBL)-producing Enterobacterales and MDR *Pseudomonas aeruginosa*. It is recommended for use when susceptibility to other first-line therapies is limited, offering a valuable option in the management of difficult-to-treat Gram-negative infections [5]. Its clinical applications are usually guided by susceptibility testing to ensure efficacy and to minimize the development of resistance.

In the intensive care unit (ICU), severe infections caused by difficult-to-treat Gram-negative pathogens, including *P. aeruginosa*, pose significant therapeutic challenges. The increasing prevalence of MDR and extensively drug-resistant (XDR) strains of *P. aeruginosa* exacerbate these challenges. Resistance mechanisms such as efflux pumps, production of β-lactamases (including carbapenemases), porin mutations, and antibiotic-modifying enzymes significantly limit the therapeutic options available. Standard antibiotics often fail against these pathogens, necessitating the use of novel agents or combination therapies, which underscore the critical need for advanced treatment options like ceftolozane/tazobactam [1,6,7,8]. Indeed, nosocomial infection rates in ICUs are high (affecting roughly 18–54% of ICU patients), with associated mortality ranging from 9% up to 60% in some reports [5]. The advent of ceftolozane/tazobactam (and similar new β-lactam/β-lactamase inhibitor combinations) offers a potential lifeline in this context by providing potent activity against many resistant Gram-negative organisms that previously had limited treatment options.

The integration of real-world data into the evaluation of ceftolozane/tazobactam is essential for enhancing our understanding of its efficacy, safety, and clinical applications, especially in a middle-income country such as Brazil [5]. While pivotal clinical trials have demonstrated its efficacy (for instance, the ASPECT-NP Phase 3 trial showed ceftolozane/tazobactam to be non-inferior to meropenem in ventilated pneumonia, with comparable clinical cure rates around 60% and similar 28-day mortality [9], observational studies offer complementary insights., Real-world studies offer invaluable insights into how ceftolozane/tazobactam performs across diverse patient populations, clinical settings, and infection severities. Real-world data can illuminate how ceftolozane/tazobactam works across varied patient profiles, infection types, and levels of illness severity that are often encountered in practice. Such studies have reported encouraging outcomes: for example, a meta-analysis of clinical studies (covering over 1600 patients) found a high overall clinical success rate of approximately 73% with ceftolozane/tazobactam (and related new agents) in treating MDR Gram-negative infections [5]. Similarly, analyses of real-world use in critical care indicate that ceftolozane/tazobactam is an effective carbapenem-sparing option, achieving clinical improvement in the majority of severe MDR cases [8]. In a Canadian registry of ICU patients with hospital-acquired and ventilator-associated pneumonia, ceftolozane/tazobactam used as directed therapy against MDR P. aeruginosa resulted in high microbiological and clinical cure rates, alongside an excellent safety profile [10]. Analyzing such data from observational studies allows clinicians to better assess the antibiotic’s true effectiveness in these complex cases, including its role in treating difficult-to-treat Gram-negative infections such as MDR *P. aeruginosa*. In addition to efficacy, real-world studies provide critical information on the safety profile of ceftolozane/tazobactam, particularly regarding adverse events in high-risk patients or in combination with other antibiotics. This data helps to refine dosing strategies, optimize treatment durations, and identify any previously unreported adverse reactions that might arise in less controlled environments. Ultimately, the relevance of real-world data lies in its ability to bridge the gap between controlled clinical studies and the practical realities of infectious disease management. By leveraging this data, healthcare providers can refine treatment guidelines, improve stewardship efforts, and enhance outcomes for patients battling severe and resistant Gram-negative infections [8].

This study was conceived to contribute to that growing real-world evidence base. Here, we describe the clinical characteristics, infection profiles, antimicrobial therapy patterns, and hospital outcomes of ICU patients in Brazil who received ceftolozane/tazobactam. Our aim is to characterize these real-world ICU experiences with ceftolozane/tazobactam and discuss the findings in light of the current literature on its effectiveness, resistance considerations, and role in managing critical infections.

## 2. Results

### 2.1. Patients’ Characteristics

Overall, 104 ICU patients were included in this study. The median age was 78 years (interquartile range [IQR] 61–87), and 43% of the patients were female. Common comorbidities were cardiovascular disease (43% of patients) and diabetes mellitus (30%), followed by dementia (20%). Notably, 21% had a history of prior pneumonia. Regarding ICU admission sources and reasons, about half of the patients (52%) were admitted directly from the emergency department, and the large majority (79%) had a medical (non-surgical) primary reason for ICU admission. In fact, infection or sepsis was the primary admission diagnosis in 61% of cases, reflecting the high illness severity of this cohort. Severity-of-illness scores at ICU entry were available for a subset (SAPS 3 was 67 [IQR 59–78] in 49 patients), and 30% had a SOFA score ≥2, indicating multi-organ dysfunction in nearly one-third. Neurologic impairment on admission was also common, with a median Glasgow Coma Score of 13 (IQR 7–15). By the time of ceftolozane/tazobactam initiation, many patients had undergone prolonged ICU care: more than half (60%) were on mechanical ventilation when therapy began, and 72% had central venous catheters in place. Invasive devices were prevalent (58% with indwelling urinary catheters and 47% with arterial lines), underscoring the critical condition and intensive monitoring of these patients (Table 1).

### 2.2. Antimicrobial Therapy

The main indication for ceftolozane/tazobactam use was pneumonia (*n* = 59, 57%), followed by urinary tract infection (*n* = 20, 19%). Pneumonia was more prevalent amongst those who were mechanically ventilated (VAP in 42 out of 62, 68%), while 16 out of 42 who were not intubated had urinary tract infections (38%). Overall, 62 patients (60%) were undergoing mechanical ventilation, 75 (72%) had central venous catheters, 60 (58%) had bladder catheters, and 49 (47%) had invasive arterial catheters. Amongst those who were mechanically ventilated, the duration of invasive respiratory support was 16 (IQR 9, 39) days.

Comparing patients that required mechanical ventilation and those that did not, the hospital length of stay at antimicrobial initiation was much longer in those who were ventilated (26 [IQR 15, 54] days) as compared with those who were not intubated (11 days IQR 3, 23). (Table 2). Ceftolozane/tazobactam was initiated empirically in the majority of patients in our cohort (*n* = 60, 57%), which did not differ between those who were intubated and not intubated. Out of the 104 patients, 44 patients had isolated pathogens in cultured clinical samples (respiratory—51%; urine—27%; blood—9%; others 13%). Out of the 44 pathogens isolated, 15 were ESBL-producing Gram-negatives (34%), 8 were carbapenem-resistant Gram-negatives (18%), 5 were Methicilin-resistant staphylococcus aureus (MRSA) (11%), and 16 were other bacteria (36%). ESBL-producing pathogens were more common in patients who were not intubated, and carbapenem-resistant Gram-negatives were more common in those who were ventilated.

Considering the overall cohort, the daily dosing of ceftolozane/tazobactam utilized was 4.5 g (47%), 9 g (44%), and 13.5 g (4%), with a duration of therapy of 10 (IQR 6, 12) days. The duration was also not influenced by the requirement of ventilation. Other antibiotics were used in the same hospital admission prior to ceftolozane/tazobactam in 88% of cases, concomitantly in 64%, and after the full course of ceftolozane/tazobactam in 58%. In ventilated patients, other concomitant antimicrobial agents were used twice as much when compared to non-intubated patients.

### 2.3. Outcomes

Clinical outcomes in this critically ill cohort were poor overall, consistent with their high illness severity. The in-hospital mortality rate was 51% (53 out of 104 patients). ICU lengths of stay and overall hospital lengths of stay were prolonged, especially among those who required mechanical ventilation. Patients who were mechanically ventilated had an ICU stay more than double that of non-ventilated patients (median 57 days [IQR 31–111] vs. 20 days [IQR 7–40], respectively). Likewise, the total hospital length of stay was substantially longer in ventilated patients (median 63 days [IQR 37–124]) compared to non-ventilated patients (35 days [IQR 17–50]). Importantly, mortality differed markedly by ventilation status: ventilated patients had a hospital mortality of 66%, which was more than twice the mortality of 29% in patients who were not intubated. This disparity highlights the grave prognosis associated with respiratory failure and ventilator-associated infections. Additionally, the ultimate disposition of survivors reflected this severity: 50% of non-ventilated patients were able to be discharged home, whereas only 29% of ventilated patients went directly home after hospitalization. Many ventilated survivors required transfer to rehabilitation or long-term care facilities, indicating substantial residual morbidity. These outcome differences are detailed in Table 3. In summary, patients requiring mechanical ventilation at the time of ceftolozane/tazobactam therapy had significantly longer and more intensive hospital courses and far higher mortality than those who were not ventilated, underscoring that they represented a more critically ill subgroup with more refractory infections (Table 3).

## 3. Discussion

Our study provides a detailed description of ICU patients undergoing antimicrobial therapy with ceftolozane/tazobactam in a real-world setting of a middle-income country. Our cohort included a large majority of elderly patients, mostly admitted to the ICU for clinical reasons, the most frequent being infectious diseases. More than half were undergoing mechanical ventilation and were admitted to the hospital for more than a week when ceftolozane/tazobactam was started. Pneumonia and urinary tract infections were the main indications for antimicrobial use, with antimicrobial therapy guided by microbiology in only 43% of cases. We found a profile of patients with long ICU and hospital lengths of stay and elevated in-hospital mortality.

These findings are contextualized within broader advancements in clinical microbiology and antimicrobial therapeutics. Seminal studies have demonstrated the critical importance of this β-lactam/β-lactamase inhibitor combination in managing severe infections [11,12,13]. Notably, its efficacy in ventilator-associated pneumonia (VAP) and infections caused by MDR and extensively drug-resistant (XDR) *P. aeruginosa* solidifies its role in combating some of the most challenging clinical pathogens. Trova Cuba et al. further emphasized the synergistic potential of combining ceftolozane/tazobactam with fosfomycin or aztreonam, demonstrating its expanded applications to carbapenem-resistant pathogens [13]. The ASPECT-NP trial serves as a cornerstone study demonstrating that ceftolozane/tazobactam offers therapeutic equivalence to meropenem in ventilated patients. The trial reported comparable clinical cure rates (60.6% vs. 57.1%) and 28-day mortality outcomes (20.1% vs. 25.8%) [12], which underscore its utility in scenarios where carbapenem resistance severely limits therapeutic choices. Carbapenem resistance continues to pose a significant challenge, especially in ventilator-associated pneumonia, as shown in our cohort (8 out of 44 isolated pathogens). Moreover, beyond VAP, ceftolozane/tazobactam has shown promise in treating complicated intra-abdominal infections and urinary tract infections. Its broad-spectrum activity, coupled with a favorable safety profile, underscores its versatility. Clinical trials have consistently highlighted its efficacy in reducing pathogen loads and improving clinical outcomes in patients with comorbid conditions or severe disease presentations. Our study cohort predominantly consisted of elderly ICU patients with severe infections, with many of them requiring prolonged hospital stays and mechanical ventilation.

Empirical initiation of ceftolozane/tazobactam was the predominant approach in this cohort, reflecting local epidemiological pressures. This contrasts with global best practice recommendations advocating microbiology-guided treatment for this antibiotic. However, the high prevalence of MDR pathogens, such as *P. aeruginosa*, in the local context, in addition to high rates of multi-organ dysfunction, may justify this empirical approach [3,7]. In contrast to data from another study from Brazil, where urinary tract infections were the main cause of infections, our cohort showed a majority of respiratory infections, especially amongst mechanically ventilated patients. Moreover, almost one third had organ dysfunction, and neurological dysfunction was frequent (septic encephalopathy), as evidenced by an altered Glasgow Coma Score. Hospital mortality was high in both cohorts [5].

The effectiveness of ceftolozane/tazobactam has been demonstrated in multiple severe Gram-negative infections, including in patients with urinary tract infections, intra-abdominal infections, and nosocomial pneumonia. Analyses of specific patient populations, including this study, have highlighted the suitability of ceftolozane/tazobactam treatment for critically ill patients, including those with comorbidities and infection with multidrug-resistant pathogens. Given the increase in the prevalence of multidrug resistance, access to alternative antibacterial agents, such as ceftolozane/tazobactam, has never been more important [10,11].

Despite the severity of illness, our data affirms that ceftolozane/tazobactam was a pivotal therapeutic option for these patients and likely contributed to whatever treatment successes were achieved. We did not specifically measure “clinical cure” in this retrospective analysis, but reports in the literature suggest that roughly two thirds of patients with MDR infections respond favorably to ceftolozane/tazobactam. For example, Neves et al. documented a ~66% favorable clinical response rate in ICU patients treated with ceftolozane/tazobactam or ceftazidime/avibactam [5], and a recent meta-analysis found about 73% clinical success with these newer β-lactam/β-lactamase inhibitors overall [10,14]. These success rates are comparable to, if not better than, those seen with older comparator agents in similar populations. Furthermore, comparative real-world data indicates that ceftolozane/tazobactam performs at least as well as other novel therapies for resistant Gram-negatives. Notably, the large multicenter CACTUS study in the USA (involving 28 hospitals) found that for MDR P. aeruginosa infections (mostly pneumonia), ceftolozane/tazobactam achieved a higher clinical success rate than ceftazidime/avibactam (61% vs. 52% at 30 days) [3]. While mortality did not differ between those treatment groups, the observed efficacy advantage and comparable safety profile of ceftolozane/tazobactam in that study reinforces its potency in real-world practice. Together with our findings, this suggests that ceftolozane/tazobactam is an effective tool for managing MDR infections in critically ill patients, yielding clinical outcomes that are on par with or superior to those of existing alternatives. It should be noted, however, that even with optimal therapy, a substantial subset of patients will not survive or be cured, due to the severity of their underlying conditions. Therefore, maximizing supportive care and source control (e.g., adequate drainage of infections) remains as important as the choice of antibiotic in influencing outcomes.

With the increasing worldwide threat of antibacterial resistance, the monitoring of pathogenic strains and continued development of novel antibacterial drugs should be considered a priority. Ceftolozane/tazobactam is an effective alternative to carbapenems, which should be used with care, taking into consideration the local microbiologic epidemiology. It is encouraging that susceptibility rates observed in real-world observational studies and clinical efficacy trials assessing ceftolozane/tazobactam in ventilated patients with pneumonia remain high; however, it is important that the emergence of non-susceptibility after treatment continues to be monitored. With the recent development and effectiveness of β-lactam/β-lactamase combinations, future guidance should consider recommendations on the preferential use of these agents in high-risk patients, where appropriate.

Emerging data suggests that ceftolozane/tazobactam may have applications beyond its current indications. Ongoing studies are exploring its efficacy in non-traditional settings, such as community-acquired infections and prophylactic use in immunocompromised patients. Additionally, advancements in rapid diagnostic tools hold the potential to further refine its application by enabling real-time pathogen identification and susceptibility testing. Future research should focus on long-term outcomes, resistance trends, post-treatment, and comparative efficacy with newer agents entering the market.

While this study provides valuable insights, several limitations must be acknowledged. Firstly, the retrospective design limits our understanding of some relevant variables studied, such as dose selection and indication of antimicrobial therapy. Also, the limited sample size hampers the interpretation and generalization of some of our findings. The lack of comparator data with other agents, such as carbapenems, limits the ability to contextualize its relative efficacy comprehensively. Additionally, the absence of data on adverse effects and clinical cure rates constrains the depth of therapeutic insights. Finally, we could not define whether the indication of ceftolozane/tazobactam was part of an escalation or de-escalation strategy, which would be important to evaluate antimicrobial stewardship practices. Future studies should address these gaps by incorporating robust methodologies and longitudinal follow-ups to capture a more holistic picture of its clinical utility.

## 4. Materials and Methods

### 4.1. Study Design, Participants, Sites, and Study Period

We performed a retrospective analysis of ICU admissions from an integrated hospital network. Data included admissions from 1 July 2018 to 28 February 2023. We included a convenience sample of all consecutive adult patients admitted to 12 private hospitals in Brazil. The Local Ethics Committee and the Brazilian National Ethics Committee (CAAE: 17079119.7.0000.5249) approved the study without the need for informed consent. All consecutive adult cases to whom ceftolozane/tazobactam was prescribed for at least 24 h from participating sites were included. Patients were excluded if there were missing data on ICU and hospital outcomes.

### 4.2. Variables, Data Collection and Quality Control

We collected anonymized data from an electronic system used for benchmarking purposes (Epimed Monitor^®^, Rio de Janeiro, Brazil) [15]. It contains information regarding the patients’ characteristics, primary diagnosis of ICU admission, severity scores such as the Simplified Acute Physiology Score 3 (SAPS-3) [16], organ support in the ICU (mechanical ventilation, vasopressor use, and renal replacement therapy), and hospital outcomes. We considered only complete cases with outcomes. Pneumonia was defined as new lung infiltrate plus clinical evidence that the infiltrate was infectious (fever, purulent secretions, leukocytosis or reduced oxygenation). Ventilator-associated pneumonia (VAP) was defined as pneumonia in patients who had been under mechanical ventilation for more than 48 h. Urinary tract infection was defined as compatible symptoms/signs of UTI with no other source *plus* urine culture showing ≥10^3^ CFU/mL. Sepsis was defined as life-threatening organ dysfunction caused by a dysregulated host response to infection, operationalized as an acute increase in the SOFA score by ≥2 points from baseline. Microbiologic data such as prescription indication, culture site, pathogen isolated, and resistance patterns were also evaluated after being retrieved from the hospital’s laboratory system, which contained the patient’s identification number and microbiology results. Multiple outcomes were analyzed, e.g., in-hospital mortality, ICU and hospital lengths of stay, and duration of mechanical ventilation. We focused on descriptive analyses. We used the median and interquartile range (IQR) for continuous variables, and frequency and proportions for categorical variables. The analyses were performed using R 4.3.2 (R Core Team (2023) [17]).

## 5. Conclusions

In summary, ceftolozane/tazobactam has demonstrated its value as a critical therapy for MDR Gram-negative infections in the ICU, showing activity against pathogens for which conventional antibiotics often fail. Our real-world analysis in Brazilian ICUs found that this antibiotic was frequently deployed empirically in elderly, severely ill patients with hard-to-treat infections (predominantly pneumonia and UTIs). The outcomes underscore both the utility and the limitations of current treatments: while ceftolozane/tazobactam likely contributed to controlling infections in many cases (as evidenced by high observed microbiological response rates in the literature and its necessity in resistant cases), the overall mortality remained high due to the patients’ critical conditions. This highlights that effective antibiotics are necessary but not sufficient on their own to save lives in this population—optimized supportive care, early source control, and prevention of healthcare-associated infections are also paramount. Moreover, the high rates of empirical use found in this cohort underscore opportunities for protocolized stewardship rather than endorsing broad empiric deployment of broad-spectrum antibiotics.

## Figures and Tables

**Table 1:** Patients’ characteristics.

Characteristic	*n* = 104
**Age (years)**	78 (61, 87)
**Sex (female)**	45 (43%)
**Comorbidities**	
Diabetes	31 (30%)
Cardiovascular disease	45 (43%)
Chronic kidney disease	17 (16%)
Immunosuppression	15 (14%)
Hematological malignancy	7 (6.7%)
Solid tumor	16 (15%)
COPD	15 (14%)
Dementia	21 (20%)
History of pneumonia	22 (21%)
**Source of admission**	
Emergency department	54 (52%)
Transfer from another hospital	18 (17%)
Other	32 (31%)
**At admission**	
Primary admission diagnosis: infection or sepsis	63 (61%)
Primary admission diagnosis: clinical	82 (79%)
SAPS 3 (*n* = 49)	67 (59, 78)
SOFA ≥ 1	39 (38%)
SOFA ≥ 2	31 (30%)
Glasgow Coma Score	13.0 (7.0, 15.0)
Mechanical ventilation	62 (60%)

COPD, chronic obstructive pulmonary disease; SAPS, Simplified Acute Physiology Score; SOFA, Sequential Organ Failure Assessment.

**Table 2:** Antimicrobial therapy characteristics according to mechanical ventilation requirements.

Characteristic	Not Intubated *n* = 42	Mechanical Ventilation *n* = 62
**Indication for antimicrobial therapy with ceftolozane/tazobactam**		
Pneumonia	17 (40%)	42 (68%)
Urinary tract infection	16 (38%)	4 (6.5%)
Skin, bone, other tissues	3 (7.1%)	3 (4.8%)
Bloodstream infection	1 (2.4%)	3 (4.8%)
Abdominal	2 (4.8%)	1 (1.6%)
Central nervous system	0 (0%)	1 (1.6%)
Other	3 (7.1%)	8 (13%)
**Hospital length of stay prior to ceftolozane/tazobactam**	11 (3, 23)	26 (15, 54)
**ICU invasive support and monitoring**		
MV duration—days (*n* = 62)	NA	16 (9, 39)
Central venous catheter	21 (50%)	54 (87%)
Bladder catheter	14 (33%)	46 (74%)
Invasive arterial catheter	2 (4.8%)	47 (76%)
**Isolated microbiology**		
Respiratory	6 (35%)	17 (61%)
Urine	8 (47%)	4 (14%)
Blood	1 (5.9%)	3 (11%)
Skin, bone, other tissues	2 (12%)	0 (0%)
Other	0 (0%)	4 (14%)
**Antimicrobial therapy characteristics**		
Daily dosing of ceftolozane/tazobactam (mg) (all 8/8 h)		
4500	24 (57%)	25 (40%)
9000	16 (38%)	30 (48%)
13,500	1 (2.4%)	3 (4.8%)
Duration of therapy (days)	9.0 (6.0, 10.0)	10.0 (7.0, 12.0)
After initial use, second course of ceftolozane/tazobactam	7 (17%)	10 (16%)
Antimicrobial therapy prior to ceftolozane/tazobactam	34 (81%)	58 (94%)
Antimicrobial therapy concomitantly with ceftolozane/tazobactam	18 (43%)	49 (79%)
Antimicrobial therapy after course of ceftolozane/tazobactam	19 (45%)	41 (66%)
**Microbiology positive**	18 (43%)	28 (45%)
**Isolated pathogens (*n* = 44)**		
ESBL producing Gram-negative pathogens	7 (44%)	8 (29%)
Carbapenem-resistant Gram-negative pathogens	1 (6.3%)	7 (25%)
MRSA	2 (13%)	3 (11%)
Others	6 (38%)	10 (36%)

ICU, intensive care unit; MV, mechanical ventilation; ESBL, Extended-Spectrum Beta-Lactamase; MRSA, Methicilin-resistant Staphylococcus aureus, NA, not available.

**Table 3:** Clinical outcomes according to mechanical ventilation requirement, isolation of Pseudomonas aeruginosa, presence of sepsis, ceftolozane/tazobactam dosage, and concomitant use of antimicrobial therapy.

Characteristic	Outcomes Evaluated		
	**Not Intubated** *n* = 42	**Mechanical** **Ventilation** *n* = 62	***p* value**
Duration of mechanical ventilation (*n* = 62)	NA (NA, NA)	16 (9, 39)	
ICU length of stay (days, IQR)	20 (7, 40)	57 (31, 111)	<0.001
Hospital length of stay (days, IQR)	35 (17, 50)	63 (37, 124)	0.002
Hospital mortality	12 (29%)	41 (66%)	<0.001
Discharged home	21 (50%)	18 (29%)	0.03
	**Others** *n* = 90	**Isolated microbe:** **Pseudomonas** *n* = 14	***p* value**
Duration of mechanical ventilation (*n* = 62)	15 (10, 40)	19 (6, 37)	0.8
ICU length of stay (days, IQR)	36 (18, 72)	77 (40, 142)	0.021
Hospital length of stay (days, IQR)	42 (26, 96)	77 (44, 184)	0.040
Hospital mortality	47 (52%)	6 (43%)	0.5
Discharged home	33 (37%)	6 (43%)	0.7
Mechanical ventilation	52 (58%)	10 (71%)	0.3
	**No sepsis** *n* = 84	**Sepsis** *n* = 20	***p* value**
Duration of mechanical ventilation (*n* = 62)	16 (10, 37)	14 (6, 46)	0.4
ICU length of stay (days, IQR)	37 (19, 80)	59 (22, 110)	0.2
Hospital length of stay (days, IQR)	44 (28, 94)	59 (36, 129)	0.3
Hospital mortality	41 (49%)	12 (60%)	0.4
Discharged home	31 (37%)	8 (40%)	0.8
Mechanical ventilation	45 (54%)	17 (85%)	0.010
	**Daily dose of C/T > 4.5 g** *n* = 55	**Daily dose of C/T = 4.5 g** *n* = 49	***p* value**
Duration of mechanical ventilation (*n* = 62)	14 (9, 37)	18 (12, 43)	0.4
ICU length of stay (days, IQR)	39 (22, 85)	39 (18, 81)	0.6
Hospital length of stay (days, IQR)	43 (28, 111)	46 (28, 108)	>0.9
Hospital mortality	28 (51%)	25 (51%)	>0.9
Discharged home	22 (40%)	17 (35%)	0.6
Mechanical ventilation	37 (67%)	25 (51%)	0.092
	**C/T alone** *n* = 37	**C/T plus concomitant antimicrobial** *n* = 67	***p* value**
Duration of mechanical ventilation (*n* = 62)	13 (10, 18)	16 (9, 40)	0.4
ICU length of stay (days, IQR)	31 (7, 49)	43 (22, 96)	0.015
Hospital length of stay (days, IQR)	37 (20, 65)	50 (32, 119)	0.023
Hospital mortality	15 (41%)	38 (57%)	0.11
Discharged home	15 (41%)	24 (36%)	0.6
Mechanical ventilation	13 (35%)	49 (73%)	<0.001

ICU, intensive care unit; NA, not available, IQR, interquertile range; C/T ceftolozane/tazobactam.

## Data Availability

Data will be provided upon reasonable request.
